# Initial Hyperleukocytosis and Neutrophilia in Nasopharyngeal Carcinoma: Incidence and Prognostic Impact

**DOI:** 10.1371/journal.pone.0136752

**Published:** 2015-09-03

**Authors:** Zhen Su, Yan-Ping Mao, Pu-Yun OuYang, Jie Tang, Fang-Yun Xie

**Affiliations:** Department of Radiation Oncology, Sun Yat-sen University Cancer Center; State Key Laboratory of Oncology in South China; Collaborative Innovation Center for Cancer Medicine, Guangzhou 510060, China; Gustave Roussy, FRANCE

## Abstract

**Background:**

This study aimed to evaluate initial hyperleukocytosis and neutrophilia as prognostic indicators in patients with nasopharyngeal carcinoma.

**Methods:**

A retrospective analysis of 5,854 patients identified from a cohort of 6,035 patients diagnosed with nasopharyngeal carcinoma was performed with initial hyperleukocytosis and neutrophilia analyzed as prognostic factors. Multivariate Cox proportional hazards analyses were applied.

**Results:**

Hyperleukocytosis was observed in 508 patients (8.7%). Multivariate analysis showed that initial hyperleukocytosis was an independent predictor of death (HR 1.40, 95%CI 1.15–1.70, *p* = 0.001), progression (HR 1.25, 95%CI 1.06–1.47, *p* = 0.007) and, marginally, distant metastasis (HR 1.21, 95%CI 0.97–1.52, *p* = 0.088). Neutrophilia was also an independent predictor of death (HR 1.46, 95%CI 1.18–1.81, *p* = 0.001), progression (HR 1.31, 95%CI 1.10–1.56, *p* = 0.003), and distant metastasis (HR 1.29, 95%CI 1.02–1.65, *p* = 0.036), after adjusting for prognostic factors and excluding hyperleukocytosis.

**Conclusion:**

Initial hyperleukocytosis and neutrophilia were independent, poor prognostic factors and may be convenient and useful biological markers for survival of patients with nasopharyngeal carcinoma.

## Introduction

Nasopharyngeal carcinoma (NPC) is a unique type of head and neck cancer with distinct pathological and clinical features that is endemic in specific populations. A high incidence (between 20–30/100,000) has been reported in areas of Southern China and Southeast Asia [1–2]. With improvements in imaging, radiotherapy techniques [3], chemotherapy and target therapy [4], survival rates have significantly improved; however, 10–20% of patients with NPC develop metastases following radical radiotherapy, and distant metastasis has become the dominant cause of treatment failure [5–6]. Therefore, it is important to identify in which cases metastasis is likely to occur. The identification of novel prognostic factors beyond the TNM stage system to identify patients at high risk is warranted.

Initial hyperleukocytosis is common in patients with solid tumors, and the incidence of hyperleukocytosis ranges from 4% to 25.6% [7]. Initial hyperleukocytosis is often accompanied by neutrophilia. Initial hyperleukocytosis or neutrophilia are indicators of poor prognosis in gynecological tumors [8–11], resected oral squamous cell carcinoma [12], anal cancer [13], metastatic colorectal cancer [14], lung cancer [15–16], bladder cancer [17], renal cell carcinoma [18], colorectal cancer [19] and gastrointestinal stromal tumors [20]. These studies showed that initial hyperleukocytosis and neutrophilia were independent prognostic factors predicting poor overall survival (OS), locoregional relapse-free survival (LRFS) and distant metastasis-free survival (DMFS) related to increased tumor burden and aggressive tumor biology [9,21].

To date, only one study has reported that pretreatment percentages of peripheral neutrophils and lymphocytes were independent prognostic factors in patients with NPC [22]. The median follow-up duration was only 41 months (range 2–60 months). Only 49 patients with stage I/II showed progression, and the authors could not explore the association between neutrophils and survival because of the small sample size. In addition, analyses of the associations between leukocytes and relapse or distant metastasis were not performed. We performed the present study to elucidate the effects of initial hyperleukocytosis and neutrophilia on the clinicopathological features of NPC and to determine whether initial hyperleukocytosis and neutrophilia were independent predictors of prognosis.

## Materials and Methods

### Ethics statement

This study was reviewed and approved by the institutional review board and ethics committee of Sun Yat-sen University Cancer Center. The study was retrospective. Patient records were anonymized and de-identified before analysis.

### Patients

We reviewed retrospectively the medical records of 6035 newly diagnosed patients from 1^st^ June 2005 to 31^st^ December 2010, with biopsy-proven, non-metastatic NPC, who were hospitalized at our center. We collected data on basic characteristics including age, gender, histological type, pretreatment hematological profile and image data. Patient records were evaluated for factors known to cause hyperleukocytosis, including evidence of an abscess or bacterial infection, acute or chronic inflammatory conditions, current corticosteroid use, and coexisting hematological malignancies. We carefully checked the blood test, urine test, feces test, chest X-ray or computed tomography, clinical manifestation (e.g. fever, rash, arthritis) and past medical history (e.g. current corticosteroid use, coexisting hematologic malignancies), especially when leukocytes were above the normal range. After exclusion of 181 patients who had other factors that cause hyperleukocytosis, 5854 patients were included in this study. All patients were restaged using the seventh edition of the AJCC/UICC Staging System for NPC [23].

The treatment strategy for all patients was based on the National Comprehensive Cancer Network Guidelines [24] and Karnofsky performance status (KPS). All patients were treated by conventional radiotherapy (CRT) or intensity modulated radiation therapy (IMRT), with or without chemotherapy. Radiation techniques and chemotherapy regimens have been described previously [25–26].

The follow-up duration was calculated from the date of first diagnosis to either the date of death or the date of last examination. OS was defined as the time from the date of first diagnosis to the date of death resulting from any cause. Progression-free survival (PFS) was defined as the time from the date of first diagnosis to the date of disease progression or death (regardless of the cause of death). LRFS and DMFS were defined as the time from the date of first diagnosis to the date of first locoregional relapse or distant metastasis, respectively.

Leukocytes and neutrophils were measured within 1 week before therapy in all patients. The number of leukocytes and neutrophils were determined using a fully automated hematology analyzer Sysmex XE-5000 (Sysmex, Kobe, Japan). Initial hyperleukocytosis was defined as a leukocyte count greater than 10×10^9^/L [27–29]. Neutrophilia was defined as a neutrophil count greater than 8×10^9^/L. Patients were separated into two groups based on the presence or absence of initial hyperleukocytosis. Patients were also separated into two groups based on the presence or absence of neutrophilia.

### Statistical analysis

The following endpoints (interval to the first defining event) were estimated: OS, PFS, LRFS and DMFS. All were analyzed using the Kaplan-Meier method and compared using log-rank tests. Multivariate analyses were performed using a Cox proportional hazards model. Chi-square (χ^2^) tests and Kruskal-Wallis H tests were used to assess the statistical significance of associations between categorical variables and the dichotomized hyperleukocytosis groups and neutrophilia groups. Two-sided *p* values less than 0.05 were considered significant. All tests were conducted using IBM SPSS version 20.0 .0 (IBM Corporation, Armonk, NY, USA).

## Results

### Baseline characteristics

A total of 5854 patients with NPC who had pretreatment blood work available were identified during the chart review. Initial hyperleukocytosis was observed in 508 patients (8.7%). Neutrophilia was observed in 407 patients (6.9%). A positive linear trend was observed from the scatter plot between leukocytes and neutrophils and Pearson’s product-moment correlation coefficient was 0.942 (*p*<0.001), indicating that the leukocytes positively correlated with the neutrophils. As shown in Table 1, initial hyperleukocytosis and neutrophilia were associated with gender, T-classification, N-classification and clinical stage (*p*<0.05 in all cases). Patients with hyperleukocytosis or neutrophilia presented a more advanced clinical stage than patients without hyperleukocytosis or neutrophilia. More male patients than female patients presented with hyperleukocytosis (9.6% *vs*. 5.8%, *p*<0.001) and neutrophilia (7.5% *vs*. 4.9%, *p* = 0.001). Among the male patients, 71.2% were at stage III/IV, 28.8% were at stage I/II. Among the female patients, 68.4% were at stage III/IV and 31.6% were at stage I/II. Advanced disease was found more frequently in male than female patients (χ^2^ = 4.039, *p* = 0.044).

**Table 1:** Demographics and treatment characteristics for patients with nasopharyngeal carcinoma.

Characteristics	ALL	Leukocyte (×10^9^/L)			Neutrophil (×10^9^/L)		
	(N, %)	Leukocyte≤10 (N, %)	Leukocyte >10 (N, %)	*p value*	neutrophil≤8 (N, %)	neutrophil>8 (N, %)	*p* value
Total	5854	5346(91.3)	508 (8.7)		5451(93.1)	403 (6.9)	
Gender				<0.001			0.001
Male	4371(74.7)	3950(73.9)	421(82.9)		4041(74.1)	330(81.9)	
Female	1483(25.3)	1396(26.1)	87 (17.1)		1410(25.9)	73 (18.1)	
Age				0.52			0.085
≤45	2997(51.2)	2723(51.1)	267(52.6)		2774(49.1)	223(55.3)	
>45	2857(48.8)	2616(48.9)	241(47.4)		2677(50.9)	180(44.7)	
T-classification				<0.001			<0.001
T1	937(16.0)	884(16.5)	53 (10.4)		894(16.4)	43 (10.7)	
T2	1279(21.8)	1179(22.1)	100(19.7)		1198(22.0)	81 (20.1)	
T3	2127(36.3)	1939(36.3)	188(37.0)		1990(36.5)	137(34.0)	
T4	1511(25.8)	1344(25.1)	167(32.9)		1369(25.1)	142(35.2)	
N-classification				0.004			0.026
N0	1133(19.4)	1062(19.9)	71 (14.0)		1071(19.6)	62 (15.4)	
N1	3314(56.6)	3012(56.3)	302(59.4)		3082(56.5)	232(57.6)	
N2	1119(19.1)	1016(19.0)	103(20.3)		1034(19.0)	85 (21.1)	
N3	288(4.9)	256(4.8)	32 (6.3)		264(4.8)	24 (6.0)	
Clinical stage				<0.001			<0.001
I	285(4.9)	272(5.1)	13 (2.6)		274(5.0)	11 (2.7)	
II	1442(24.6)	1342(25.1)	100(19.7)		1363(25.0)	79 (19.6)	
III	2395(40.9)	2190(41.0)	205 (40.4)		2241(41.1)	154(38.2)	
IV	1732(29.6)	1542(28.8)	190(37.4)		1573(28.9)	159(39.5)	
Pathologic types				0.803			0.128
I	115(2.0)	107(2.0)	8 (1.6)		111(2.0)	4 (1.0)	
II	321(5.5)	293(5.5)	28 (5.5)		304(5.6)	17 (4.2)	
III	5418(92.6)	4946(92.5)	472(92.9)		5036(92.4)	382(94.8)	
Radiotherapy				0.746			0.847
CRT	4013(68.6)	3668(68.6)	345(67.9)		3735(68.5)	278(69.0)	
IMRT	1841(31.4)	1678(31.4)	163(32.1)		1716(31.0)	125(31.0)	
Chemotherapy				<0.001			0.001
No	1084(18.5)	1025(19.2)	59 (11.6)		1035(19.0)	49 (12.2)	
IC	1282(21.9)	1158(21.7)	124(24.4)		1190(21.8)	92 (22.8)	
CC	1761(30.1)	1617(30.2)	144(28.3)		1646(30.2)	115(28.5)	
IC+CC	1472(25.1)	1313(24.6)	159(31.3)		1350(24.8)	122(30.3)	
CC+AC	255(4.4)	233(4.4)	22 (4.3)		230(4.2)	25 (6.2)	

CRT: conventional radiotherapy; IMRT: intensity modulated radiation therapy; IC: induced chemotherapy; CC: concurrent chemotherapy; AC: adjuvant chemotherapy

The median survival time for all patients was 55.9 months (range: 3.1 to 119.2 months). 9.7% (567/5854) of patients developed locoregional relapse, 12.9% (757/5854) developed distant metastases and 15.4% (901/5854) of patients died. The five-year survival rates for the patient population were: OS 84.6% (95%CI 83.6%–85.6%); PFS 74.7% (95%CI 73.5%–75.7%); LRFS 89.4% (95%CI 88.4%–90.4%); and DMFS 86.1% (95%CI 85.1%–87.1%).

### Survival analysis

**Fig 1:**
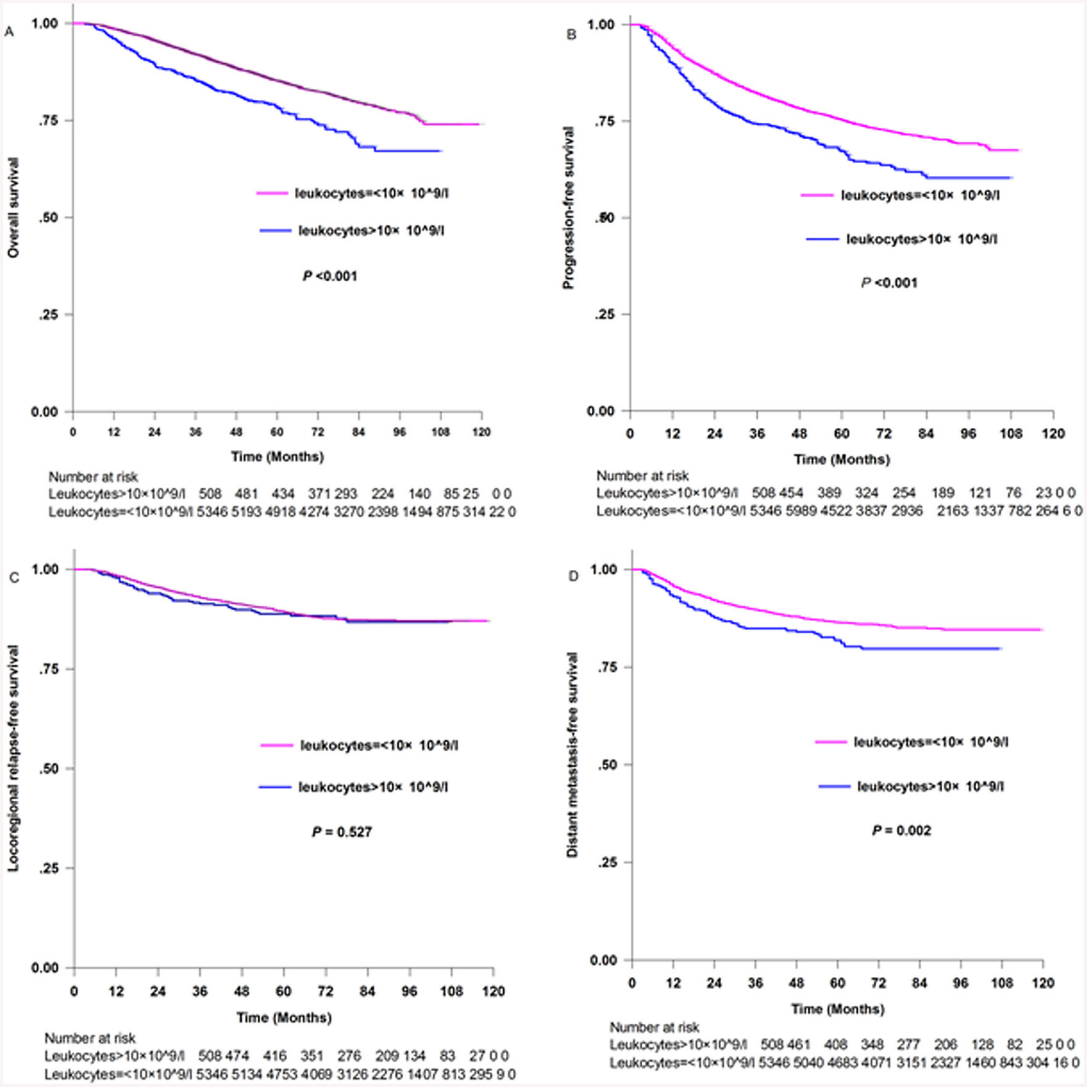
Kaplan-Meier survival curves for patients with leukocytes>10×10^9/l and patients with leukocytes = <10×10^9/l. (A) Overall survival. (B) Progression-free survival. (C) Locoregional relapse-free survival and (D) Distant metastasis-free survival.

Fig 1 shows the Kaplan-Meier estimates of patients with and without initial hyperleukocytosis. The five-year survival rates of patients with initial hyperleukocytosis compared with those without hyperleukocytosis, respectively, were: OS [78.4% (95%CI 74.5%–82.3%) and 85.20% (95%CI 84.2%–86.2%), *p*<0.001, Fig 1A]; PFS [67.2% (95%CI 62.9%–71.5%) and 75.4% (95%CI 74.2%–76.6%), *p*<0.001, Fig 1B]; LRFS [88.8% (95%CI 85.7%–91.9%) and 89.5% (95%CI 88.3%–90.3%), *p* = 0.527, Fig 1C]; and DMFS [81.9% (95%CI 78.2%–85.6%) and 86.5% (95%CI 85.5%–87.5%), *p* = 0.002, Fig 1D].

**Fig 2:**
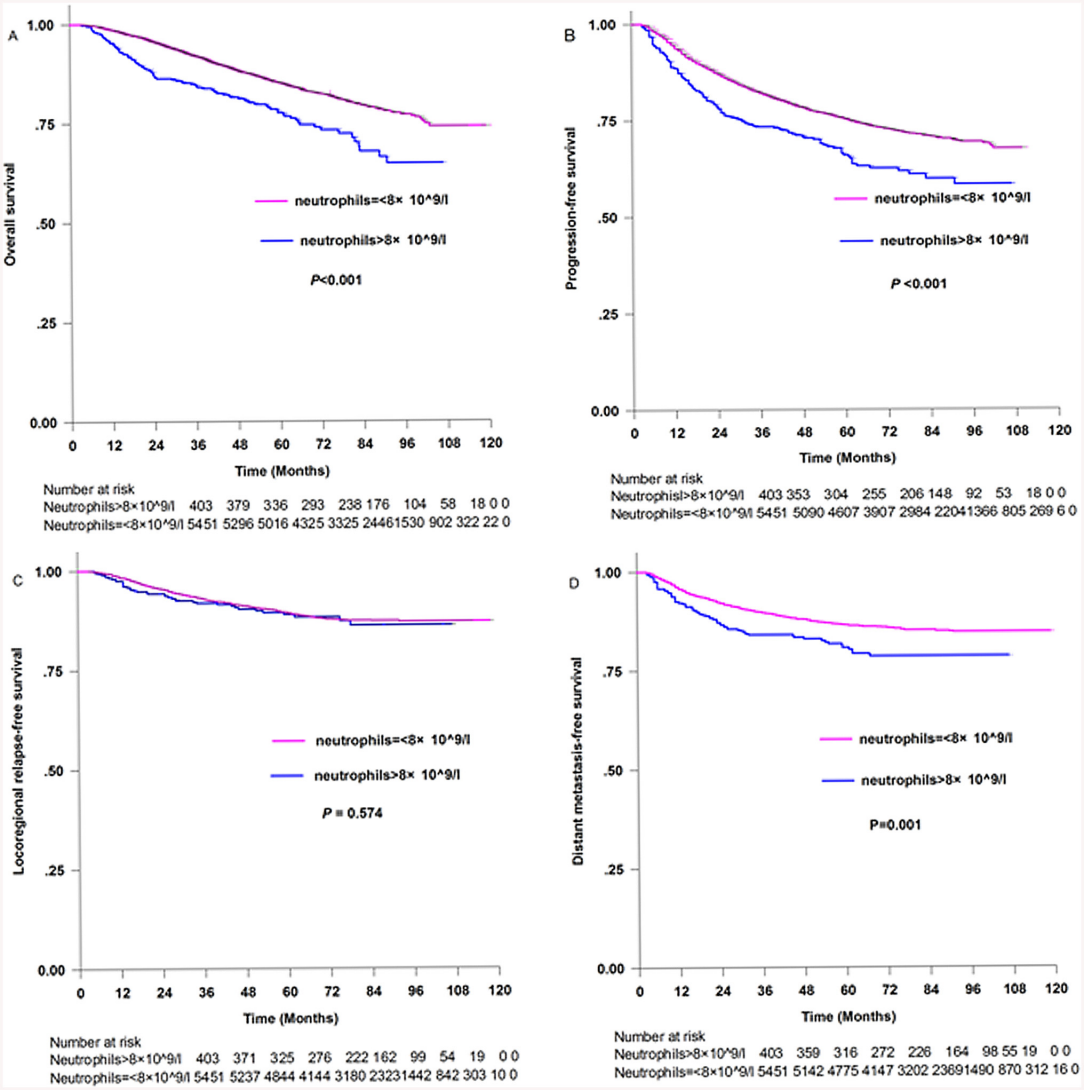
Kaplan-Meier survival curves for patients with neutrophils>8×10^9/l and patients with neutrophils = <8×10^9/l. (A) Overall survival. (B) Progression-free survival. (C) Locoregional relapse-free survival and (D) Distant metastasis-free survival.

Fig 2 shows the Kaplan-Meier estimates of patients with and without neutrophilia. The five-year survival rates for patients with neutrophilia compared with those without neutrophilia, respectively, were: OS [77.6% (95%CI 73.1%–82.1%) and 85.1% (95%CI 84.1%–86.1%), *p*<0.001, Fig 2A]; PFS [65.9% (95%CI 60.8%–70.1%) and 75.4% (95%CI 74.2%–76.6%), *p*<0.001, Fig 2B]; LRFS[88.9% (95%CI 85.4%–92.6%) and 89.5% (95%CI 88.3%–90.3%), *p* = 0.574, Fig 2C]; DMFS [80.8% (95%CI 76.7%–84.9%) and 86.5% (95% CI 85.5%–87.5%), *p* = 0.001, Fig 2D].

The significant predictors for survival of NPC are summarized in Table 2. In univariate analysis, initial hyperleukocytosis, neutrophilia, sex, T stage and N stage were significantly associated with OS, PFS and DMFS (Table 2, all *p*<0.05). Evaluated as a continuous variable, leukocytes were also associated with an increased risk of death (HR 1.09, 95%CI 1.05–1.12, *p*<0.001), progression (HR 1.07, 95%CI 1.04–1.09, *p*<0.001), relapse (HR 1.04, 95%CI 1.00–1.09, *p* = 0.03) and distant metastasis (HR 1.06, 95%CI 1.02–1.09, *p* = 0.001) (Table 2). When evaluated as a continuous variable, neutrophils were also associated with an increased risk of death (HR 1.11, 95%CI 1.07–1.14, *p*<0.001), progression (HR 1.09, 95%CI 1.06–1.11, *p*<0.001), relapse (HR 1.04, 95%CI 1.00–1.09, *p* = 0.052) and distant metastasis (HR 1.08, 95%CI 1.04–1.12, *p*<0.001) (Table 2).

**Table 2:** Univariate and multivariate analyses of survival for all patients with nasopharyngeal carcinoma.

Endpoint	Variable	Univariate analysis		Multivariate analysis	
		HR (95%CI)	*p*	HR (95%CI)	*p*
OS	Leukocyte				
	>10 *vs* ≤10	1.63 (1.34–1.97)	<0.001	1.40 (1.15–1.70)	0.001
	continuous	1.09 (1.05–1.12)	<0.001	1.05 (1.02–1.08)	0.002
	Neutrophil				
	>8 *vs* ≤8	1.68 (1.36–2.07)	<0.001	1.46(1.18–1.81)	0.001
	continuous	1.11 (1.07–1.14)	<0.001	1.07 (1.03–1.11)	<0.001
	Sex	0.60 (0.51–0.71)	<0.001	0.65 (0.55–0.77)	<0.001
	Age				
	>45 *vs* ≤45	1.91 (1.67–2.19)	<0.001	1.93 (1.69–2.21)	<0.001
	T stage	1.55 (1.45–1.67)	<0.001	1.50 (1.40–1.61)	<0.001
	N stage	1.82 (1.68–1.97)	<0.001	1.79 (1.66–1.94)	<0.001
PFS	Leukocyte				
	>10 *vs* ≤10	1.45 (1.23–1.70)	<0.001	1.25 (1.06–1.47)	0.007
	continuous	1.07 (1.04–1.09)	<0.001	1.03 (1.01–1.06)	0.007
	Neutrophil				
	>8 *vs* ≤8	1.51 (1.26–1.79)	<0.001	1.31 (1.10–1.56)	0.003
	continuous	1.09 (1.06–1.11)	<0.001	1.05 (1.02–1.08)	<0.001
	Sex	0.64(0.56–0.73)	<0.001	0.69(0.60–0.78)	<0.001
	Age				
	>45 *vs* ≤45	1.32 (1.19–1.47)	<0.001	1.32 (1.19–1.46)	<0.001
	T stage	1.42 (1.34–1.50)	<0.001	1.38 (1.30–1.46)	<0.001
	N stage	1.64 (1.53–1.74)	<0.001	1.61 (1.51–1.71)	<0.001
LRFS	Leukocyte				
	>10 *vs* ≤10	1.10 (0.82–1.46)	0.528	0.98 (0.74–1.31)	0.918
	continuous	1.04 (1.00–1.09)	0.03	1.02 (0.98–1.06)	0.399
	Neutrophil				
	>8 *vs* ≤8	1.10 (0.80–1.51)	0.574	1.01 (0.97–1.06)	0.528
	continuous	1.04 (1.00–1.09)	0.052	0.99 (0.71–1.36)	0.934
	Sex	0.66 (0.54–0.82)	<0.001	0.69 (0.56–0.84)	<0.001
	Age				
	>45 *vs* ≤45	1.15 (0.97–1.35)	0.101	1.14 (0.97–1.35)	0.111
	T age	1.31 (1.20–1.42)	<0.001	1.29 (1.18–1.40)	<0.001
	N age	1.35 (1.21–1.50)	<0.001	1.33 (1.20–1.47)	<0.001
DMFS	Leukocyte				
	>10 *vs* ≤10	1.43 (1.14–1.79)	0.002	1.21 (0.97–1.52)	0.088
	continuous	1.06 (1.02–1.09)	0.001	1.02 (0.98–1.05)	0.294
	Neutrophil				
	>8 *vs* ≤8	1.52 (1.20–1.94)	0.001	1.29 (1.02–1.65)	0.036
	Continuous	1.08 (1.04–1.12)	<0.001	1.04 (1.01–1.08)	0.034
	Sex	0.65 (0.54–0.78)	<0.001	0.68 (0.57–0.82)	<0.001
	Age				
	>45 *vs* ≤45	1.07 (0.93–1.24)	0.342	1.07 (0.93–1.23)	0.361
	T age	1.49 (1.38–1.61)	<0.001	1.45 (1.34–1.57)	<0.001
	N age	1.96 (1.80–2.14)	<0.001	1.92 (1.76–2.09)	<0.001

HR: unadjusted hazard ratio; CI: confidence interval; OS: overall survival; PFS: progression-free survival; LRFS: locoregional relapse-free survival; DMFS: distant metastasis-free survival.

Multivariate analyses adjusted for age group (≤45 and >45 years-old), gender, T-classification (T1/T2/T3/T4), N-classification (N0/N1/N2/N3), Pathological types, type of radiotherapy and type of chemotherapy.

After adjusting for covariates, multivariate analysis (not including neutrophilia) confirmed that initial hyperleukocytosis was independently associated with an increased risk of death (HR 1.40, 95%CI 1.15–1.70, *p* = 0.001), progression (HR 1.25, 95%CI 1.06–1.47, *p* = 0.007) and distant metastasis (HR 1.21, 95%CI 0.97–1.52, *p* = 0.088) (Table 2). In addition, leukocytes evaluated as a continuous variable were also independently associated with an increased risk of death (HR 1.05, 95%CI 1.02–1.08, *p* = 0.002) and progression (HR 1.03, 95%CI 1.01–1.06, *p* = 0.007) (Table 2).

In multivariate analysis (not including initial hyperleukocytosis), neutrophilia was independently associated with an increased risk of death (HR 1.46, 95%CI 1.18–1.81, *p* = 0.001), progression (HR 1.31; 95%CI 1.10–1.56, *p* = 0.003) and distant metastasis (HR 1.29, 95%CI 1.02–1.65, *p* = 0.036) (Table 2). In addition, neutrophils evaluated as a continuous variable were also independently associated with an increased risk of death (HR 1.07, 95%CI 1.03–1.11, *p*<0.001), progression (HR 1.05, 95%CI 1.02–1.08, *p*<0.001) and distant metastasis (HR 1.04, 95%CI 1.01–1.08, *p* = 0.034) (Table 2).

In multivariate analysis, including both hyperleukocytosis and neutrophilia, neutrophilia remained significant for poorer OS (HR 1.46, 95%CI 1.18–1.81, *p* = 0.001), PFS (HR 1.31, 95%CI 1.10–1.56, *p* = 0.003) and DMFS (HR 1.29, 95%CI 1.02–1.65, *p* = 0.036).

### Stratified analysis by clinical stage

We carried out an exploratory stratified analysis by clinical stage. The associations between initial hyperleukocytosis, neutrophilia and survival are summarized in Table 3. Significant associations of initial hyperleukocytosis with PFS were observed in both groups of patients with high clinical stage III/IV (HR 1.21, 95%CI 1.01–1.44, *p* = 0.037) and low clinical stage I/II (HR 1.55, 95%CI 1.03–2.33, *p* = 0.036). A significant association between initial hyperleukocytosis with OS was observed only in patients with stage III/IV (HR 1.39, 95%CI 1.13–1.71, *p* = 0.002). Significant associations of neutrophilia with PFS (HR 1.29, 95%CI 1.07–1.56, *p* = 0.009) and OS (HR 1.73, 95%CI 1.59–1.89, *p*<0.001) were only observed in patients with high clinical stage (III/IV). In addition, when evaluated as a continuous variable, leukocytes were associated with an increased risk of death (HR 1.05, 95%CI 1.01–1.08, *p* = 0.006) and progression (HR 1.03, 95%CI 1.01–1.06, *p* = 0.032) only in patients with stage III/IV. Neutrophils evaluated as a continuous variable were associated with an increased risk of death (HR 1.07, 95%CI 1.03–1.11, *p*<0.001) and progression (HR 1.05, 95%CI 1.02–1.08, *p* = 0.003) in patients with stage III/IV and were associated with an increased risk of progression (HR 1.08, 95%CI 1.01–1.16, *p* = 0.020) in patients with stage I/II.

**Table 3:** Multivariate analysis of survival for patients stratified by clinical stage.

Endpoint	Variable	I/II		III/IV	
		HR (95%CI)	*p*	HR (95%CI)	*p*
OS	Leukocyte				
	>10 *vs* ≤10	1.48 (0.83–2.63)	0.185	1.39 (1.13–1.71)	0.002
	continuous	1.06 (0.97–1.16)	0.176	1.05 (1.01–1.08)	0.006
	Neutrophil				
	>8 *vs* ≤8	1.23 (0.62–2.44)	0.548	1.73 (1.59–1.89)	<0.001
	continuous	1.06 (0.96–1.17)	0.241	1.07 (1.03–1.11)	<0.001
PFS	Leukocyte				
	>10 *vs* ≤10	1.55 (1.03–2.33)	0.036	1.21 (1.01–1.44)	0.037
	continuous	1.06 (0.99–1.13)	0.061	1.03 (1.01–1.06)	0.032
	Neutrophil				
	>8 *vs* ≤8	1.43 (0.90–2.26)	0.127	1.29(1.07–1.56)	0.009
	continuous	1.08 (1.01–1.16)	0.02	1.05 (1.02–1.08)	0.003
LRFS	Leukocyte				
	>10 *vs* ≤10	1.31 (0.71–2.45)	0.39	0.92 (0.66–1.27)	0.594
	continuous	1.05 (0.97–1.15)	0.23	1.01 (0.96–1.05)	0.748
	Neutrophil				
	>8 *vs* ≤8	1.17 (0.57–2.40)	0.672	0.95 (0.66–1.36)	0.756
	continuous	1.08 (0.99–1.19)	0.095	0.99 (0.95–1.05)	0.908
DMFS	Leukocyte				
	>10 *vs* ≤10	1.80 (0.98–3.31)	0.059	1.16 (0.91–1.47)	0.237
	continuous	1.05 (0.95–1.16)	0.292	1.01 (0.98–1.05)	0.464
	Neutrophil				
	>8 *vs* ≤8	1.84 (0.95–3.55)	0.07	1.03 (0.99–1.07)	0.101
	continuous	1.10 (0.99–1.22)	0.071	1.24 (0.96–1.61)	0.099

HR: unadjusted hazard ratio; CI: confidence interval; OS: overall survival; PFS: progression-free survival; LRFS: locoregional relapse-free survival; DMFS: distant metastasis-free survival.

Multivariate analyses adjusted for age group (≤45 and >45 years-old), gender, T-classification (T1/T2/T3/T4), N-classification (N0/N1/N2/N3), Pathological types, type of radiotherapy and type of chemotherapy.

Collectively, these results indicated that initial hyperleukocytosis and neutrophilia are important prognostic factors for survival among patients with NPC.

## Discussion

Initial hyperleukocytosis and neutrophilia are frequently observed in the clinic. The frequencies of initial hyperleukocytosis and neutrophilia in our study were comparable to previous clinical studies [7–8,10–13]. However, the different criteria for the diagnosis of hyperleukocytosis and neutrophilia must be considered. The definition of hyperleukocytosis across these studies was remarkably consistent, defined as a leukocyte count >10×10^9^/L in all cases, except for two studies: one of endometrial carcinoma [10], which used a leukocyte count>8.2×10^9^/L; and one of recurrent cervical cancer [11], which used a leukocyte count>9×10^9^/L. The definition of neutrophilia across these studies varied, ranging from 3.9×10^9^/L to 7.5×10^9^/L [18,20,30–31]. The probable reason is that the criteria for test instruments varied in different centers. In our study, the normal range of neutrophils was determined between 2×10^9^/L and 8×10^9^/L. We therefore chose a cut-off point of 8×10^9^/L.

In our study, we found that initial hyperleukocytosis and neutrophilia had significant impacts on the risk of death, progression and metastasis. Our findings are somewhat similar to previous studies [8,11,18,20]. He et al. reported that pretreatment NLR (neutrophil to lymphocyte) and percentages of lymphocytes and neutrophils were independent prognostic factors; however, counts of neutrophils and lymphocytes were not associated with OS [22]. In a stratified analysis, significant associations of NLR and percentages of neutrophils and lymphocytes with PFS occurred only among patients with high clinical stage (III/IV), but not those with low clinical stage (I/II). However, in our study, a significant association of leukocytes with PFS was observed among both groups of patients, indicating that initial hyperleukocytosis as a predictor of survival for patients with NPC was effective in patients with high and low clinical stage. Initial hyperleukocytosis reflects tumor burden to a certain extent. Our study also found that neutrophilia was an independent prognostic indicator of distant metastasis. In addition, as continuous variables, leukocytes and neutrophils were still independent prognostic factors for survival for patients with NPC. Our study was sufficient to prove the associations, not only because of the larger sample size (5854 patients *vs*. 1410 patients), but also because of the longer median follow-up [56 months (range: 3.1 to 119.2 months) *vs*. 41 months (range: 2 to 60 months)]. Furthermore, initial hyperleukocytosis may be a sign of a leukemoid reaction, which is described as an increase in leukocyte count. Cvitkovic et al. first reported the leukemoid reaction as a new clinical symptom associated with NPC [32]. In 2014, Kus et al. also reported a case of a leukemoid reaction associated with pediatric NPC [33]. In Cvitkovic’s study, it was hypothesized that a leukemoid reaction could be important in the diagnosis and follow-up of patients with NPC: its appearance and reappearance being the first manifestation of malignancy or the relapse. Therefore, initial hyperleukocytosis and neutrophilia may be convenient and intuitive markers for diagnosis and follow-up of NPC.

Based on our findings, we proposed several potential explanations for the poor survival associated with initial hyperleukocytosis and neutrophilia. The cause of hyperleukocytosis in patients with cancer vary, including infection, corticosteroids, intoxication, severe hemorrhage, bone marrow metastases, paraneoplastic leukemoid syndrome and use of granulocyte-colony stimulating factor (G-CSF) [21,34]. We tried to exclude patients who had hyperleukocytosis caused by these other factors before analysis. Initial hyperleukocytosis and neutrophilia were associated with solid tumor burden [9,21]. Our results showed that initial hyperleukocytosis and neutrophilia correlated with gender, T-classification, N-classification and clinical stage, suggesting that patients with initial hyperleukocytosis or neutrophilia were more likely to be at an advanced disease stage and, therefore, have poorer survival. Furthermore, initial hyperleukocytosis or neutrophilia not only indicated advanced disease stage, but also could be markers of aggressive biological behavior, indicating increased risk of invasive and distant metastasis. In multivariate analysis, initial hyperleukocytosis or neutrophilia remained independent prognostic indicators of survival. In patients with hyperleukocytosis, treatment failures were not associated with clinical stage; in contrast, in patients without hyperleukocytosis, treatment failures were significantly associated with clinical stage [28]. Initial hyperleukocytosis or neutrophilia could be a sign of aggressive cancer. This suggested that initial hyperleukocytosis or neutrophilia might be an indicator of more need for chemotherapy in patients with the same clinical stage. Donskov and colleagues summarized the role of initial neutrophilia in cancers in a review, and found that an elevated neutrophil count was a strong and independent risk factor for poorer outcomes, and that increasing the dose of cytokines, chemotherapy, or targeted therapy did not eliminate the negative prognostic impact [35]. In other words, high baseline tumor-related neutrophilia might prevent a proportion of patients from benefiting from therapy. Patients with neutrophilia might need greater intensity of treatment compared with patients without neutrophilia at the same clinical stage.

In solid tumors, upregulation of hematopoietic growth factors, such as G-CSF/ granulocyte-macrophage colony-stimulating factor (GM-CSF), promotes tumor progression [36]. Concomitant expression of G-CSF and its cognate receptor G-CSFR was observed in multiple epithelial cancers, which are the mostly poorly differentiated and invasive [37–39]. Mabuchi et al. observed a significantly stronger immunoreactivity for G-CSF in tumors obtained from patients with hyperleukocytosis (48/50) than in tumors obtained from patients without hyperleukocytosis (10/203; *P*<0.0001) [28]. A G-CSF receptor-mediated increase in β1-integrin expression has been proposed to cause increased adhesion and invasiveness of these carcinoma cells, which could promote metastasis [40]. The production of G-CSF by tumor cells could, therefore, account for the diffuse leukocyte or neutrophil infiltration of the tumor and could also account for the lower DMFS.

Neutrophils, as the first line of defense in the immune system, play an important role in anti-tumor activity by releasing tumor-cytotoxic substances or by activating other anti-tumor immune effector cells [41–42]. They also have a significant impact on tumor angiogenesis and immunosuppression, as well as migration, invasion and metastasis. Neutrophils are emerging as central players in the inflammatory tumor microenvironment. During chronic cancer-related inflammation they appear to promote tumor growth by influencing key processes of tumor initiation and progression [43–47]. Essentially, initial hyperleukocytosis or neutrophilia indicate advanced disease stages and aggressive biological behavior. Initial hyperleukocytosis or neutrophilia play important roles in tumor progression by altering the tumor microenvironment.

A limitation of the present study was its retrospective nature. Although a thorough chart review was conducted to identify and exclude patients with other potential causes of hyperleukocytosis and neutrophilia, the inherent limitations of a retrospective review mean that some patients may have been wrongly included in the analysis. In addition, this study collected data of patients from 2005 to 2010. Over that five-year period, the measurement standards of instruments are likely to have undergone some minor changes. We considered normal or abnormal counts as study variables.

In conclusion, we showed that initial hyperleukocytosis and neutrophilia in patients with NPC are significantly associated with poor prognosis in terms of OS and PFS. Neutrophilia is also significantly correlated with poor DMFS. Initial hyperleukocytosis and neutrophilia are independent, poor prognostic factors and might be convenient and useful biological markers for survival of patients with NPC. Further work to validate these findings should include the evaluation of serum and tumor colony-stimulating factors, which may provide therapeutic targets for immune-modulating strategies to improve survival in patients with NPC.

## Supporting Information

S1 Table The original data of the study. (XLS)
